# Can digital governance stimulate green development? Evidence from the program of “pilot cities regarding information for public well-being” in China

**DOI:** 10.3389/fpubh.2024.1463532

**Published:** 2025-02-04

**Authors:** Xuxin Zou, Jiadi Min, Shuang Meng, Wenguan Dai, Xinyang Li

**Affiliations:** ^1^School of Economics, Beijing Wuzi University, Beijing, China; ^2^School of International Trade and Economics, Central University of Finance and Economics, Beijing, China; ^3^Renmin Business School, Renmin University of China, Beijing, China

**Keywords:** digital governance, green development, public well-being, industrial structure, green innovation

## Abstract

Digital governance is an important goal of government digital transformation, and the policy of “pilot cities regarding information for public well-being” in China is an important initiative to promote the reform of government governance system and realize green development. Based on the panel data of 282 cities in China from 2007 to 2020, this paper analyzes the mechanism and path of the impact of digital governance on green development using the program of “pilot cities regarding information for public well-being” as a quasi-natural experiment. The double-difference model and mediation effect model are utilized. The results show that digital governance can significantly promote green development, and the path of influence is the supererogation and the rationalization of industrial structure, i.e., digital governance can promote the green development of the cities by enhancing the supererogation and the rationalization of industrial structure. In addition, there is obvious heterogeneity in the impact of digital governance on green development. The policy exerts a stronger effect on economically developed cities than on economically backward cities, and a stronger effect on large cities than on small- and medium-sized cities. Therefore, the relevant departments should continue to optimize the pilot policy regarding information for public well-being, strive to improve the government’s digital governance, focus on economically backward cities and small-sized cities, actively cultivate the green development, promote the optimization and upgrading of industrial structure, and vigorously support cross-regional exchanges and cooperation, so as to jointly achieve green and high-quality development.

## 1 Introduction

Green development refers to promoting economic growth while minimizing resource consumption and environmental pollution in order to achieve sustainable development of the economy, society and the ecosystem. It emphasizes the realization of harmonious coexistence between human beings and nature through the adoption of clean energy, energy-saving technologies and ecological protection measures (1). With the increasingly serious conditions of global climate change, green development has progressively attracted extensive attention from governments and all sectors of society. The United States government has enacted the Clean Power Plan, which aims to gradually reduce the proportion of coal-fired power generation by encouraging the use of natural gas and renewable energy, and to promote cleaner ways of producing electricity; the European Union has issued the European Green Deal, which plans to promote the development of clean energy, improve energy efficiency, and reduce greenhouse gas emissions in a number of areas, such as energy, buildings, agriculture and transportation.

As for developing countries, India has issued a National Action Plan to Combat Climate Change, which proposes to develop solar energy, improve energy efficiency, develop sustainable agriculture, etc., aiming at reducing the impact of climate change and promoting green development; and Brazil has adopted a National Climate Change Policy focusing on the protection of the Amazon rainforest, reduction of deforestation activities, and sustained reduction of greenhouse gas emissions. As the world’s largest developing country, China has also been actively promoting green development in recent years, and has made it a key objective of its ongoing reform of its governmental governance system. The wave of green innovation has led to the development of energy-saving and clean technologies, which use less energy than traditional products. Similarly, green technologies in transportation reduce the consumption of fossil fuels. This reduction in energy consumption helps to reverse the process of environmental degradation. Therefore, this reduction in carbon emissions has had a positive impact on public health (2).

Theoretically, reform of the government governance system can prompt the government to more effectively use policy tools, such as tax incentives, subsidies, and green finance, to incentive enterprises and industries to transition to green and further realize green development. Moreover, digital governance can enable governments to shorten the approval time for green patents and simplify the approval process, which can help increase the willingness of enterprises to undergo green transformation and green innovation, thereby reducing environmental pollution and promoting good public health (3). In reality, then, does the reform of government governance structure truly promote green development? What are the mechanisms and paths of this process? Addressing these questions is of great theoretical and practical significance for further deepening the reform of the government governance system and promoting high-quality development.

With the development of digital technology, the digital transformation of the government has become an important aspect of the reform of the government governance system. In this context, the Central Committee of the Communist Party of China and the State Council of China have successively formulated and issued a series of documents to guide the government’s digital transformation work in order to improve the government’s digital governance capabilities, such as the “State Council’s Guiding Opinions on Strengthening the Construction of Digital Government” “Guidelines for the Construction of a National Integrated Government’s Big Data System” among others. It should be noted that, in the first half of 2014, the National Development and Reform Commission and other 12 departments jointly issued the “Notice on Accelerating Implementation of the Program regarding Information for Public Well-being” and “Notice on Agreeing to the Construction of National Pilot Cities regarding Information for Public Well-being in 80 Cities such as Shenzhen.” This is an important measure for deepening the reform of the government governance system and promoting digital transformation of the government. It is proposed that through the implementation of this program, each pilot city should be promoted to break the “information island,” strengthened the interconnection of resources and information among various departments, and realized the deep integration of information technology with the application of people’s livelihood, then continuously improving the digital level of government governance, and finally achieving the goal of assisting people and enterprises.

Furthermore, on the basis of summarizing the experience of these pilot cities, the General Office of the State Council of China forwarded the “Implementation Plan of Pilot Cities regarding Information for Public Well-being by ‘Internet + Government Services’ “, which is formulated by the National Development and Reform Commission and other 10 departments in April 2016. This plan proposed to cover 80 national pilot cities in various provinces (districts and cities) and establish a unified comprehensive government service window, data sharing platform, and government service information system, realizing the “one application, one window, one network” of various government service matters, and forming replicable experiences that are gradually implemented throughout the country. In other words, implementation of this policy will improve the level of government digital governance and make it easier for people to benefit from better public services and obtain social information. Additionally, pilot projects were selected from 80 cities to accumulate experience and reduce costs. After nearly 10-years of in-depth reform, China’s digital governance capacity has attained leapfrog development. According to the e-government survey report released by the United Nations, the rank of e-government in China has improved from 78th in 2012 to 43rd in 2022. China has become one of the countries with the highest growth in the world and took the first level in the e-government development index (EGDI).

As the government’s digital transformation is advancing swiftly, scholars have also conducted substantial studies on digital governance. First, there have been many studies on the definition of a concept. Digital governance, also called electronic governance, is a new governance model in the digital age (4). The concept of digital governance is divided into two levels: broad and narrow. From a broad perspective, digital governance refers to the organization and operation of society with the support of digital technology, including the comprehensive management of economic and social resources (5); from a narrow perspective, digital governance refers to a governance model that simplifies the process of government administration and public affairs and improves the degree of democratization by using digital information technology in the government’s interaction with civil or economic society and the government’s internal operation (6).

The current study of digital governance largely focuses on the description of characteristics and practical experience (7). Although some studies have employed case analysis (8), comparative analysis (9), and other approaches, theoretical research and practice of digital governance remain out of touch because of the lack of actual data and causal inference methods. In the healthcare sector, digital governance should use user-centered design (10). This approach prioritizes the needs and preferences of patients and healthcare providers, ensuring that digital tools are intuitive and accessible. By involving users in the design process, healthcare systems can enhance engagement, improve usability, and ultimately lead to better health outcomes.

Additionally, user-centered design fosters trust and satisfaction, encouraging more effective communication and collaboration between patients and providers. In the field of environmental governance, digital governance can establish collaborative platforms and formulate relevant policies tailored to local conditions (11). Digital governance can also help the government establish smart digital platforms and monitoring and management systems through big data, cloud computing and other technologies to predict the trend of epidemic diseases and achieve disease early warning.

Moreover, research on digital governance is largely concentrated on the subject of public management, including the impact of digital governance on political trust (12), anti-corruption (13), and other elements. Particularly in developing countries, a significant number of scholars have studied the role of digital governance in public administration. For example, Kumar et al. (14) find that digital governance reduces government corruption in the delivery of public services by studying the Rural Internet Project in Tamil Nadu, India; Rotta et al. (15) find that digital governance contributes to a sustainable developmental relationship between authorities and citizens by studying 903 municipal websites across Brazil; Mutula et al. (16) find, using the South African government as the subject of their study, that digital governance improves openness and transparency in government bidding processes. In addition, digital governance can help to promote free and fair elections.

In addition to the field of public administration, some scholars have focused their research on regional development and economic growth, such as Duan et al. (17). Through theoretical research, they argue that digital technology is favorable to urban governance innovation, which guides the urban governance system toward modernization and fosters high-quality urban growth. Kwilinski et al. (18) used panel data from 25 countries to verify that the digitalization of government has a U-shaped impact on inclusive economic growth by creating a canonical cointegrating regression model. Lv et al. (19) integrated the rural grassroots comprehensive information management system, incorporating the concept of green ecology into the rural governance process. This allows rural areas to better enjoy the benefits brought by the new management model, thereby promoting prosperity in these regions.

In conclusion, there is a large amount of research on digital governance in the existing literature, but most of it stays at the stage of theoretical analysis and lacks research on the relationship between digital governance and green development. In fact, from the perspective of the theory of planned behavior, digital governance can influence public and corporate attitudes toward green development through data transparency and information disclosure, e.g., opening up environmental data and disseminating successful cases of green development can enhance people’s positive perceptions of environmental protection behaviors, which in turn prompts them to adopt green behaviors; whereas, at the level of institutional theory, enterprise green transformation is the key to achieve green development and do a good job in public health (20), digital governance can be digitally enforced through the implementation of laws and regulations, such as the online environmental monitoring, electronic regulatory platform, from the aspect of external institutional environment to force enterprises to comply with the requirements of green development, thus promoting the realization of green development. In addition, in terms of empirical testing. Although some researchers have empirically analyzed the impact of digital governance on economic and social development, they have mainly focused on individual areas such as economic growth or residents’ income, and have not carried out in-depth studies on green development.

In view of this, this paper’s marginal contributions are mainly in three aspects. First, this study takes the “pilot cities regarding information for public well-being” project as a quasi-experiment, analyzes the panel data of 282 cities in China from 2007 to 2020, and empirically evaluates the mechanisms and paths of digital governance’s impact on green growth based on theoretical analyses, so as to fill in the gaps existing in existing researches in this regard. Second, The DID model is particularly effective in this context as it controls for unobserved factors that may influence the results. By examining the changes in outcomes before and after the policy implementation in both groups, we can isolate the causal effect of the policies. This approach helps to account for any pre-existing trends and provides a clearer understanding of the policies’ impact on the targeted outcomes. By designating pilot cities as the experimental group, we can effectively assess the impact of the implemented policies. Third, this paper studies the impact of digital governance on green development from the perspective of digital governance. Compared with the existing literature that only broadly explores the impact of digital economy on green development, the perspective is more specific, and the conclusions drawn follow more closely to the actual situation, and can directly provide opinions and suggestions for improving the relevant policies.

## 2 Research hypotheses

### 2.1 The direct impact of digital governance on green development

The program of “pilot cities regarding information for public well-being” aims to deeply integrate information technology and government governance, and enhance the government’s digital governance capabilities, thereby improving government service efficiency, optimizing resource allocation, reducing market failure, and promoting economic and social development. This program plays a vital role in fostering the green growth of the city, which can be seen in the following three areas.

First, the program has changed the situation of information asymmetry. Information asymmetry is a prevalent problem in the market, that is, the information received by buyers and sellers is different in the transaction. In the sphere of environmental protection, citizens may not have enough information on green products or services, so they may not have enough choice of environmental protection. This program aims to integrate all kinds of isolated and scattered public service resources by using digital technology to provide citizens with more accurate information, so as to eliminate this information asymmetry (21) and encourage people to purchase more green products. At the same time, it can avoid the information asymmetry between the central government and local governments, strengthen the central government’s environmental supervision over local governments, reduce the negative actions of local governments profiting from pollution, and thus promote the green development of the economy (22).

Second, the implementation of this program meets the demands of consumers on public goods such as environmental protection information. Environmental information can be viewed as public goods because once the information is provided to a person, others can also acquire the same information for free. Therefore, private firms may be unwilling to devote a lot of resources to provide such public goods, since it is difficult for them to profit from selling such information. Thus, government actions are vital. This program aims to realize the equality of essential public services, which is actually a form of public goods provided by the government (23). By providing environmental information as a public benefit, the government may meet the public’s need for environmental information, which can raise the environmental awareness of citizens and enhance the green growth of the city.

Third, this program has a beneficial externality effect on green development. Externality refers to a market failure; that is, market prices fail to reflect the impact of products or services on third parties (non-trading parties). Environmental protection behavior typically involves certain externalities; in other words, the environmental protection behavior of an entity or individual will have a certain influence on third parties, but this impact is not reflected by market prices. This program can help citizens better understand the externality of environmental protection behavior by using information technology to eliminate the gap between various departments and individuals, thus enhancing their motivation for environmental protection. For example, by publicizing information on enterprises’ environmental protection behavior, it is easier for financial institutions to provide green credit for well-performing enterprises to support their development, and for tax departments to provide preferential policies such as tax subsidies to encourage their development, thus promoting the green and low-carbon transformation and development of the whole city. In addition, the government conveys environmental consciousness to people by providing policy information (24), which also helps mold the accurate concept of environmental protection for citizens, thus improving the positive externalities of urban green growth. In summary, this study proposes the following hypotheses:

*H1*: Digital governance can promote green development.

### 2.2 The mediating effect of optimization of industrial structure on the relationship between digital governance and green development

This program is based on digital technology through the construction of government information resource sharing and exchange mechanisms to accomplish cross-level and cross-departmental information sharing and business collaboration. In this process, the government can release more power to the public, social organizations, and market players and solve the problem of information asymmetry and incompleteness between the government and market players through digital governance. Therefore, the impact of policy uncertainty on corporate investment behavior and business decision-making is lessened, and the optimization and upgrading of industrial structures can be realized (25). Furthermore, the optimized and upgraded industrial structure usually focuses on the effective use and recycling of resources, while green industry often adopts more environmentally friendly production technology and resource utilization methods, which can reduce the waste of resources.

Digital governance enables the government to better guide policies and provide financial support, encouraging enterprises to engage in green innovation for products and services and develop more environmentally friendly alternatives. It also promotes the adoption of eco-friendly technologies and processes to reduce waste and emissions, enhancing overall environmental standards within the industry. Additionally, by adjusting industrial layouts (26), digital governance can facilitate the clustering of green industries, creating a green industrial chain and ecological network that fosters regional sustainable development. Therefore, the optimization and upgrading of industrial structures will help promote the green growth of the city. In summary, this study proposes the following hypothesis:

*H2*: Digital governance can promote green development by optimizing industrial structure.

## 3 Research design

### 3.1 Model specification

The model employed in this study was based on the DID method, which is widely used for policy evaluation (27). An advantage of the DID model is its ability to mitigate the selection bias and influence of external factors to a certain extent. To study the impact of digital governance on green development, this study uses a program implemented in 2014 as a quasi-experiment and employs a DID model for empirical analysis. The core idea of the DID model is to compare changes in an indicator between two groups: the experimental group (program cities) and control group (non–program cities). By computing the difference in the indicator before and after policy implementation for both groups, the model can estimate the effect of the policy on the indicator. The rationale for this is that the “pilot cities regarding information for public well-being” policy marks a major reform in the field of digital governance in China, and provides an opportunity for a natural experiment to study the impact of digital governance on green development. The double difference model can effectively control the time effect before and after the implementation of the policy and the potential difference between the treatment group and the control group, so as to accurately assess the causal effect of the policy. Through this method, the specific impact of digital governance on green development can be clearly identified, and the influence of other interfering factors can be excluded to a certain extent. The specific model is defined in Equation 1.


(1) $$GD_{\mathrm{it}}=a_{0}+\alpha_{1}DID_{\mathrm{it}}+\mathrm{\alpha Control}s_{\mathrm{it}}+\mu_{i}+\nu_{t}+\varepsilon_{\mathrm{it}}$$

where the subscripts i and t represent cities and years, respectively. $GD_{\mathrm{it}}$ is the green development level of the city in year, $DID_{\mathrm{it}}$ is the interaction term of the treatment group dummy variable and the time dummy variable affected by the program, and $\mathrm{Control}s_{\mathrm{it}}$ is the other factor affecting green development. $\mu_{i}$ denotes the city-fixed effect, $\nu_{t}$ refers to the time-fixed effect, and $\varepsilon_{\mathrm{it}}$ is a stochastic disturbance term assumed to be independently and identically distributed (i.i.d.).

### 3.2 Variables selection

#### 3.2.1 Dependent variable

##### 3.2.1.1 Green development (GD)

Based on the research methods of Zou et al. (28), the reason for this is that energy efficiency measures the amount of energy consumed per unit of GDP, and a smaller value means that the city consumes less energy to produce the same economic value, which is directly related to the sustainability of the city.

As for the measurement method of energy efficiency, considering the availability of energy consumption data of prefecture-level cities, this paper mainly adopts three kinds of energy sources to measure the energy consumption of prefecture-level cities, namely, the total amount of natural gas supply, the total amount of liquefied petroleum gas supply, and the total electricity consumption of the whole society, which are converted into coal consumption. Among them, the natural gas discounted standard coal factor is 1.33 kg standard coal/meter, the LPG discounted standard coal factor is 1.7143 kg standard coal/kg, and the electricity discounted standard coal factor is 0.1229 kg standard coal/kWh. The specific calculation method is: firstly, these three kinds of energy are converted into standard coal (10,000 tons); secondly, the sum of the three kinds of energy (10,000 tons) is divided with the total annual GDP of the locality, and thus the amount of energy consumed per unit of GDP, that is, energy efficiency.

#### 3.2.2 Independent variable

##### 3.2.2.1 Digital governance (DID)

This binary variable indicates whether a city is part of the program of “Pilot Cities regarding information for public well-being.” Its value was set as follows:

For the pilot cities of the program, was set to 1 in the initial year, they were chosen as pilot cities and remained at 1 for all subsequent years, while it was set to 0 for all other years.

For the non-pilot cities, did remains at 0 across all years.

#### 3.2.3 Control variables

To accurately assess the impact of digital governance on green development, it is necessary to control for other factors affecting green development. In view of this, this study adds five control variables to the model, including human capital, government intervention, foreign investment intensity, transportation facilities, and scientific research support, in accordance with established practices in the existing literature (29, 30).

##### 3.2.3.1 Human capital (HC)

The ratio of college students to local residents serves as an indicator of Human capital. This indicator was chosen because well-educated individuals are more aware of the importance of environmental protection. They are more inclined to adopt environment-friendly technologies and sustainable production methods to effectively use resources, reduce energy consumption and waste, and promote green development.

##### 3.2.3.2 Government intervention (GI)

The ratio of government fiscal expenditure to regional GDP is utilized as a measure of government intervention. This indicator was chosen because moderate government intervention can help promote green development. However, if government intervention is too high, it may violate market principles and hinder green development.

##### 3.2.3.3 Foreign investment intensity (FII)

The ratio of foreign direct investment to regional GDP represents the intensity of foreign investment. This indicator was chosen because foreign investment is usually accompanied by the introduction of advanced technology and management experience, which helps cities use environmentally friendly and sustainable production technologies to promote green development.

##### 3.2.3.4 Transportation facilities (TF)

Transportation facilities are expressed in terms of the number of road miles per unit area. Transportation is one of the major sources of energy consumption and greenhouse gas emissions. The number of road miles per unit area reflects the level of transportation infrastructure, which directly affects the efficiency of the movement of people and goods in a city. Good transportation facilities can reduce traffic congestion and improve travel efficiency, thereby reducing fuel waste and pollution emissions caused by vehicles stalled on the road.

##### 3.2.3.5 Scientific research support (SRS)

The ratio of government science expenditures to total fiscal expenditures denotes scientific research support. This indicator was chosen because high scientific research support is helpful in promoting scientific and technological innovation, particularly in the research and development of green technology. By investing in scientific research, cities can promote the development and application of environmental protection technologies and increase the level of local green development.

#### 3.2.4 Mediating variables

##### 3.2.4.1 Industrial structure supererogation (ISS) and industrial structure rationalization (ISR)

Considering that the optimization of industrial structure mainly includes the supererogation and rationalization level of industrial structure, this study uses the ratio of the added value of the tertiary industry to the added value of the primary and secondary industries as a measure of the industrial structure supererogation (ISS), and employs the deviation degree of industrial structure as a measure of the industrial structure rationalization (ISR). The formula for the degree of industrial structure deviation is shown in Equation 2.


(2) $$SR=\overset{3}{\underset{i=1}{\sum }}\frac{Y_{i}}{Y}|\frac{Y_{i}/L_{i}}{Y/L}-1|=\overset{3}{\underset{i=1}{\sum }}\frac{Y_{i}}{Y}|\frac{Y_{i}/Y}{L_{i}/L}-1|$$

Where i denotes industry, Y refers to industrial added value, L represents the number of employees, and i =1, 2, and 3 denote the first, second, and third industries, respectively.

According to the hypothesis of classical economics, the productivity level of each industrial sector is the same, that is, $Y_{i}/L_{i}=Y/L$ , when the economy reaches equilibrium. The industrial structure deviation is equal to zero, indicating that the industrial structure has reached a reasonable state. In other words, the deviation degree of the industrial structure is a negative index, which means that the smaller the value, the more reasonable is the industrial structure. In addition, this study takes the reciprocal of the index and then takes the logarithm as the measurement index of the industrial structure, that is, ISR= $\ln\left(1/SR\right)$ . The larger the index after conversion, the more reasonable is the industrial structure.

### 3.3 Data collection and descriptive statistics

Considering the difficulty of obtaining data in some remote areas, this research employs data from 282 cities in China from 2007 to 2020, which are sourced from publications such as the CN Deep Data, China Urban Statistical Yearbook, provincial statistical yearbooks, and various statistical yearbooks of individual cities. Some missing values were supplemented using interpolation. The descriptive statistics of the relevant variables are presented in Table 1.^1^

**Table 1:** Descriptive statistics of variables.

Variable	Mean	SD	Min	Median	Max	Obs
GD	0.126	0.171	0.008	0.094	6.177	3,948
DID	0.126	0.332	0.000	0.000	1.000	3,948
HC	0.017	0.020	0.000	0.010	0.128	3,948
GI	0.188	0.102	0.043	0.162	1.027	3,948
FII	0.018	0.021	0.000	0.012	0.353	3,948
TF	1.037	0.495	0.047	0.997	2.628	3,948
SRS	0.016	0.016	0.001	0.011	0.207	3,948
ISS	0.732	0.399	0.094	0.645	5.164	3,948
ISR	−0.539	1.425	−7.918	−0.515	5.241	3,948

Source: China City Statistical Yearbook, China Environment Yearbook, and China Energy Yearbook.

The results in the table show that the average value of green development level is 0.126. The maximum value is 6.177. The minimum value is 0.008. The standard deviation is 0.171. Thus, the green development levels of different regions are highly varied. Additionally, there are obvious distinctions in human capital, government intervention, foreign investment, environmental regulation, and scientific research support in different locations.

## 4 Results

### 4.1 Baseline test

As this study selects more variables, it is necessary to test whether multicollinearity exists between variables before regression. By calculating the variance inflation factor (VIF) of each variable, it is found that all are less than three, indicating that there is no serious multicollinearity between the variables. To ensure the robustness of the regression results, firstly, the individual fixed effect and time fixed effect variables are added for regression, and then, the control variables are added for regression. Table 2 presents the results.

**Table 2:** Baseline test results.

	1	2
Variable	GD	GD
DID	−0.040***	−0.035**
	(0.014)	(0.014)
HC		−0.166
		(0.498)
GI		0.257***
		(0.0858)
FII		−0.128
		(0.111)
TF		0.012
		(0.025)
SRS		−0.597**
		(0.262)
Year FE	YES	YES
City FE	YES	YES
Cons	0.123***	0.092***
	(0.005)	(0.027)
*N*	3,948	3,948
*R* ^2^	0.093	0.102

(1) Standard errors in parentheses; (2) * *p* < 0.1, ** *p* < 0.05, *** *p* < 0.01.

The results show that digital governance has always had a strong positive impact on green development, regardless of whether the control variables are included. These findings substantiate the validity of Hypothesis 1.

In addition to digital governance, the level of government intervention significantly inhibited urban green development, while the level of research support significantly promoted urban green development. Specifically, for every 1% increase in the level of government intervention, the level of urban green development will decrease by 0.257%, while for every 1% increase in the level of research support, the level of urban green development will increase by 0.597%. The possible reasons for this are: on the one hand, excessive government intervention may lead to non-market allocation of resources, resulting in resource waste and inefficiency. Especially in the field of green development, too much administrative intervention may lead to misallocation of resources and inhibit the motivation of market players to innovate independently. On the other hand, government intervention in the market through subsidies, protection or direct intervention may lead to enterprises’ dependence on the government rather than improving efficiency and promoting technological innovation through market competition. This dependence may weaken the innovation drive of enterprises, which in turn inhibits their green development.

In addition, scientific research support provides the necessary financial, equipment and talent support for the research and development of green technologies, thus promoting the birth of new technologies and processes. And technological innovation is the core driving force of green development, which can significantly improve resource utilization efficiency and reduce pollution emissions. Moreover, through the support of scientific research, the results of innovation can be more quickly and widely applied to actual production, thus promoting the green development of the city. For example, new environmental protection technologies and clean energy technologies can be rapidly popularized in cities through the promotion and application of enterprises, thus promoting the overall level of green development (31).

It should be noted that the effects of human capital, foreign investment and transportation facilities are not significant, possibly because, first, while the ratio of the number of students enrolled in higher education to the local resident population may reflect the quantity of human capital input to some extent, the quality of human capital is equally important. Even if there are a large number of school students, if the quality of training and education is not high, it may not be able to adequately improve the quality of the labor force, thus affecting its role in green development; secondly, although the increase in the intensity of foreign investment can contribute to a certain extent to the green development, if foreign investment is concentrated in energy-intensive and high-energy-consumption industries, it may need to invest a large amount of time and resources in the transformation and upgrading of industrial structure, thus slowing down the city’s development and the development of the city. Upgrading, thus slowing down the pace of the city’s green transformation; third, increasing road mileage usually facilitates the use of private cars, however, the extensive use of private cars may lead to increased energy consumption and carbon emissions, which is contrary to the goal of green development. Even if road density increases, the increase in road mileage may not contribute to green development if there is no shift in transportation modes toward low-carbon and environmentally friendly modes.

### 4.2 Robustness checks

To ensure the robustness of the baseline model and to verify the stability of the findings, a series of robustness tests were conducted. The purpose of these robustness tests is to strengthen the credibility of the conclusions of the study.

#### 4.2.1 Parallel trend test

**Figure 1:**
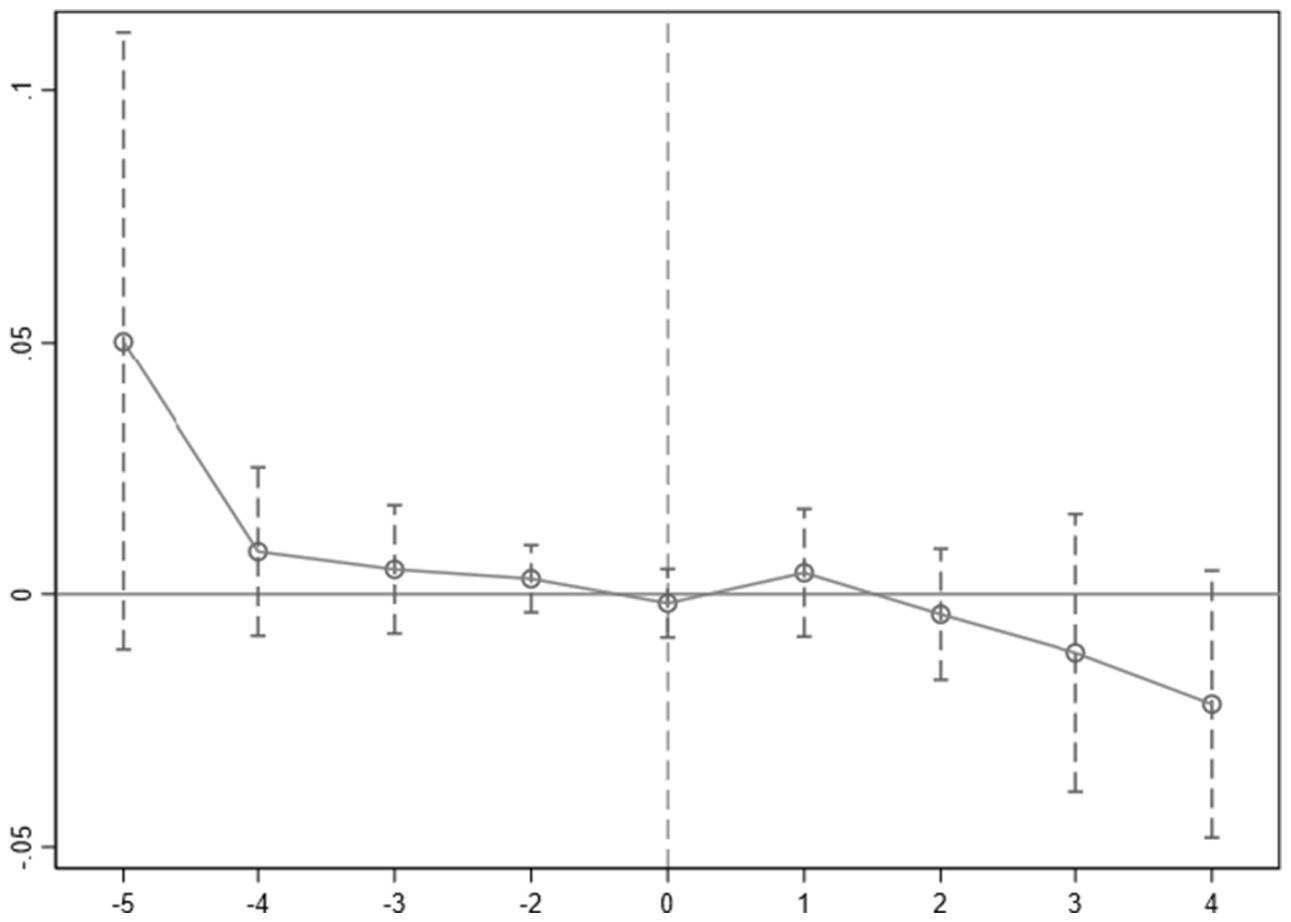
Parallel trend test result.

When applying the DID methodology, the parallel trend assumption must be met to ensure the validity of the results. The parallel trends test seeks to determine whether the groups’ trajectories are parallel in the absence of the program’s effect. In other words, there is no significant difference in the green development level between the experimental and control groups before program implementation. Based on the research methods of Alder et al. (32) and Roth (33), this study used the event analysis method to test whether the parallel trend assumption is valid. The specific model is set in Equation 3.


(3) $$GD_{\mathrm{it}}=\tau +\overset{4}{\underset{j=-5}{\sum }}\chi_{j}\mathrm{Polic}y_{i,t-j}+\mathrm{\psi Control}s_{\mathrm{it}}+\mu_{i}+\nu_{t}+\varepsilon_{\mathrm{it}}$$

where *policy* is a dummy variable that indicates the presence of the pilot program in a specific year, denoted as year t-j, which takes the value of 1 when the program is implemented in that year, and 0 otherwise. The results are depicted in Figure 1.

The results indicate that there was no significant difference between the experimental and the control groups before the implementation of the program. Consequently, the parallel trend test was successfully conducted, affirming the fulfillment of the essential prerequisite for the DID model.

#### 4.2.2 PSM-DID test

**Figure 2:**
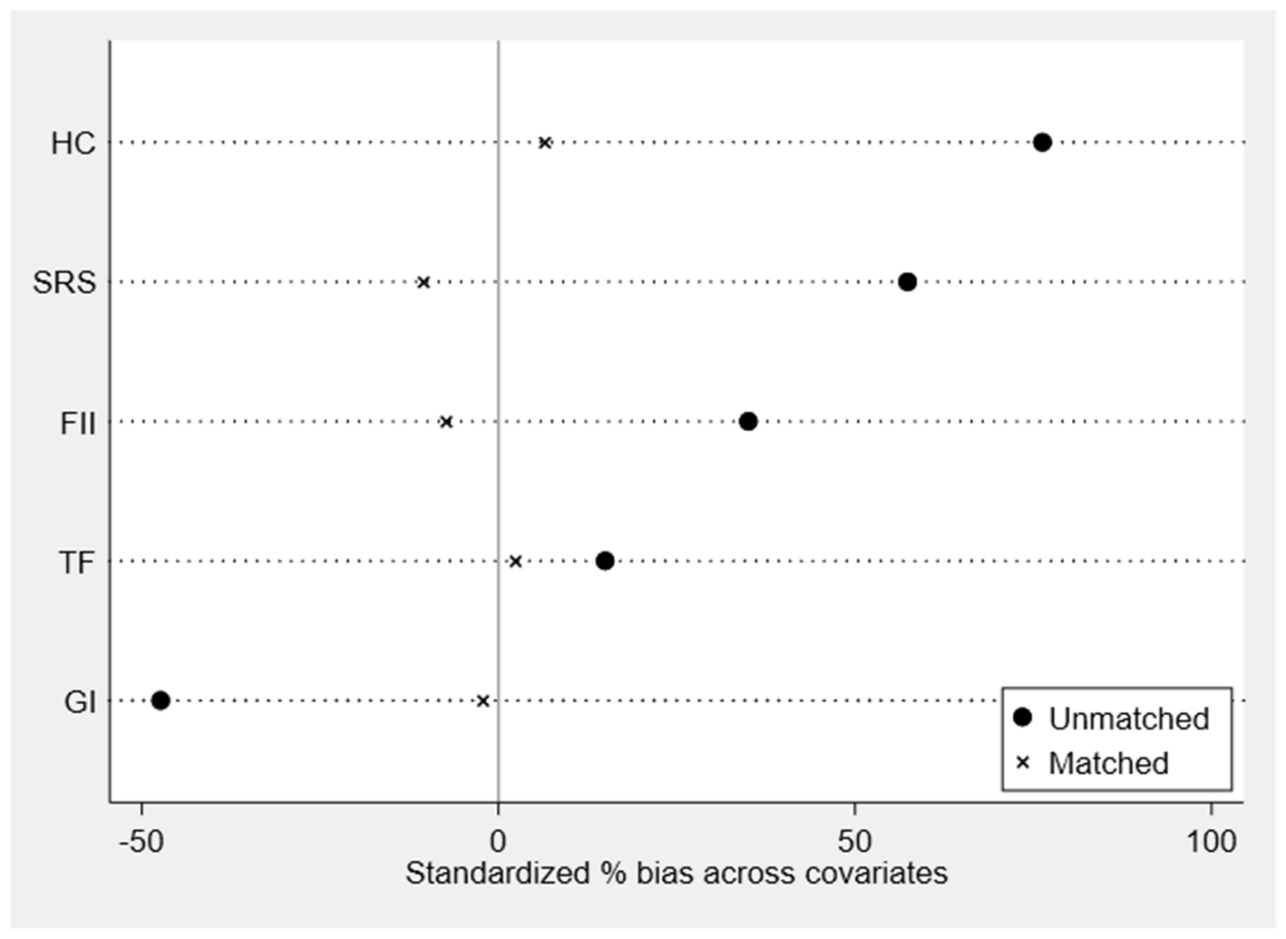
Equilibrium test of the covariates.

To mitigate the potential bias arising from this nonrandom assignment of pilot cities, the study employed the Propensity Score Matching (PSM) method with nearest neighbor matching before applying the DID methodology. To assess whether the matched samples adhere to the equilibrium hypothesis in the PSM matching, we conducted a test to evaluate whether there were significant differences in the matching covariates between the treatment and control groups. The results of this test are presented in Figure 2.

The results in Figure 2 show that after matching, the difference between the experimental and control groups is greatly reduced, indicating that the PSM-DID method can be used to test the effect of digital governance. It should be noted that this study uses the method of “nearest neighbor matching” to match, and the matched data are introduced into Equation 1 for regression. Column 1 of Table 3 presents the regression results. It can be seen that after matching, digital governance still has a significant positive impact on green development, which verifies the baseline regression results.

**Table 3:** Robustness checks results (1).

	1	2	3	4	5
	PSM-DID test	Double-headed tail processing	Excluding other policy interference		
Variable	GD	GD	GD	GD	GD
DID	−0.033**	−0.015***	−0.033***	−0.031*	−0.034**
	(0.014)	(0.005)	(0.013)	(0.016)	(0.014)
Smart City Pilot			−0.013		
			(0.011)		
Broadband China Pilot				−0.012	
				(0.013)	
Low-carbon City Pilot					−0.002
					(0.012)
Controls	YES	YES	YES	YES	YES
Year FE	YES	YES	YES	YES	YES
City FE	YES	YES	YES	YES	YES
Cons	0.083***	0.084***	0.092***	0.093***	0.092***
	(0.028)	(0.014)	(0.027)	(0.027)	(0.028)
*N*	3,902	3,948	3,948	3,948	3,948
*R* ^2^	0.100	0.497	0.102	0.102	0.102

(1) Standard errors in parentheses; (2) *p < 0.1, **p < 0.05, ***p < 0.01.

#### 4.2.3 Double-headed tail processing

To eliminate the influence of extreme values, this research performs a 5% double-headed tail reduction on continuous variables, and then performs regression again. The results are shown in Column 2 of Table 3. It can be seen that after dealing with the extreme values, digital governance still significantly promotes green development at the 5% level, which is consistent with the baseline regression results.

#### 4.2.4 Excluding other policy interference

In order to exclude other policies that may affect green development during the timeframe from 2009 to 2020, this study control the “smart pilot program,” “broadband China pilot program” and “low-carbon pilot” program, respectively, (34). Columns 3, 4, and 5 in Table 3 show the regression results, respectively. It can be seen that after adding the three aforementioned variables, the regression coefficient of digital governance remains significantly positive, thereby bolstering the validity of the baseline model’s argumentation.

#### 4.2.5 Replacing the DID variable with lagged values for one period and two periods

To eliminate endogeneity effects, we use the lagged DID variables for one period and two periods as instrumental variables for robustness tests. The results are shown in Table 4. It can be seen that the F-statistic in the first stage is greater than 10 and significant at the 1% level, indicating that there is no issue with weak instrumental variables. Moreover, after controlling for endogeneity, the impact of digital finance on economic resilience and security remains positively significant, consistent with the baseline regression results.

**Table 4:** Robustness checks results (2).

	1	2	3	4
Variable	DID	GD	DID	GD
DID		−0.043^***^		−0.055^***^
		(0.099)		(0.014)
L.DID	0.771^***^			
	(0.027)			
L2.DID			0.534^***^	
			(0.032)	
Controls	YES	YES	YES	YES
Year FE	YES	YES	YES	YES
City FE	YES	YES	YES	YES
Cons	0.525^***^	0.914^***^	1.004^***^	1.009^***^
	(0.121)	(0.165)	(0.170)	(0.175)
*N*	3,692	3,666	3,408	3,384
F-first stage	840.28***		279.995***	

(1) Standard errors in parentheses; (2) *p < 0.1, **p < 0.05, ***p < 0.01.

#### 4.2.6 Placebo test

**Figure 3:**
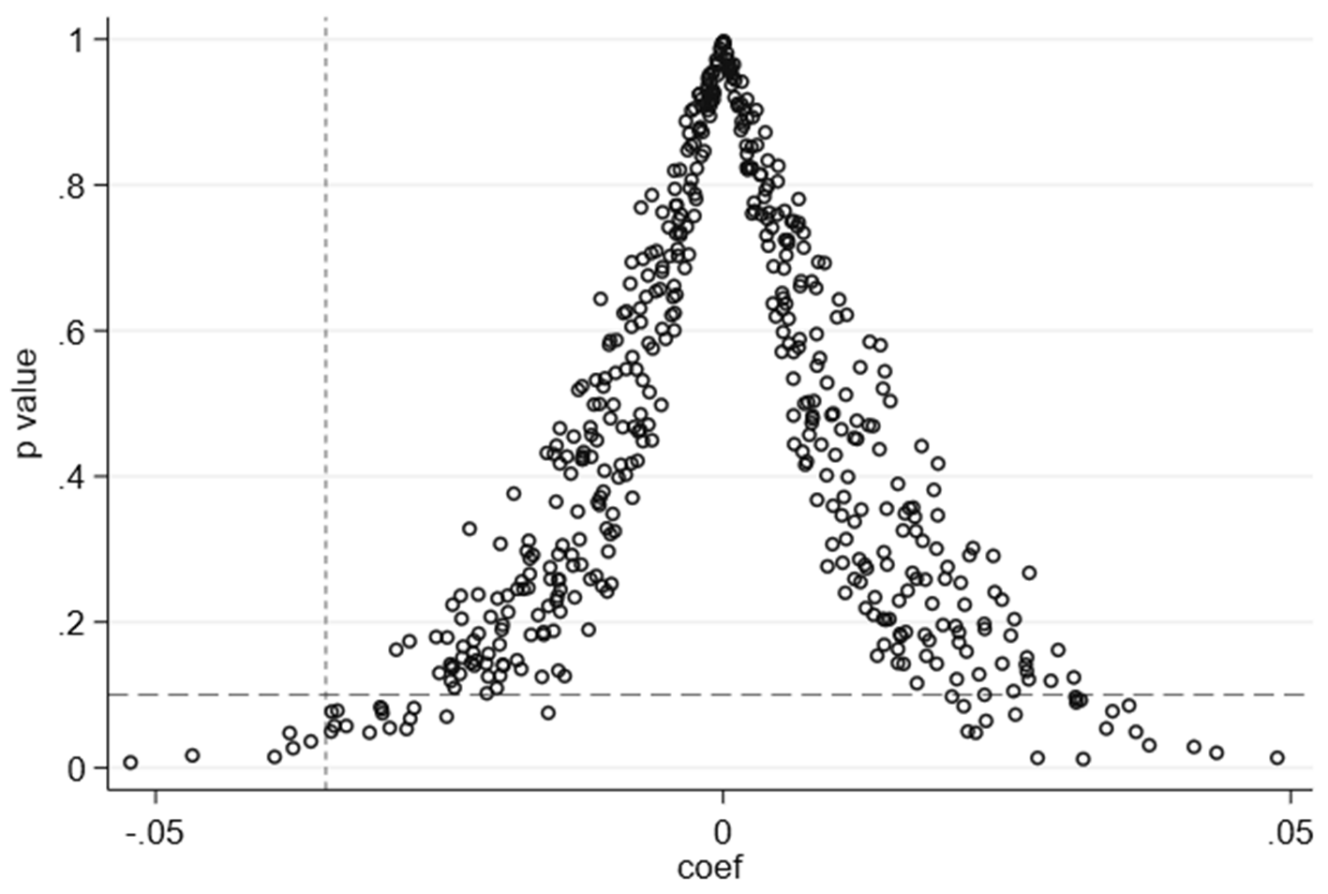
Placebo test results.

Despite the inclusion of essential control variables in the baseline model, the potential for omitting significant factors exists, which could introduce bias into the test results. To counteract the influence of these unaccounted factors on green development, we adopted a placebo test approach and conducted a placebo test by randomizing the treatment and control groups. Specifically, we randomly designated an equivalent number of cities as pilot cities and the remaining as non–pilot cities of the program for a particular year. This random assignment is repeated 500 times. The results are presented in Figure 3.

The results show that the coefficients closely align with a normal distribution with a mean of zero. Moreover, a significant majority of their associated *p*–values surpass the 0.1 threshold. This pattern of results signifies a substantial deviation from the outcomes of the baseline model. Consequently, this placebo test indicates that random sampling does not impact green development, thereby proving the robustness of the baseline regression results.

### 4.3 Heterogeneity analysis

It has been verified that the construction of pilot cities regarding information for public well-being significantly stimulates green development. However, China is a vast territory, with prominent heterogeneity among cities in terms of location advantage, economic development level, population size, natural resources, and human educational levels. Therefore, this study conducted a heterogeneity analysis with the aim of delving deeper into the variations in the effects of the program on green development based on local economic development levels, and population sizes. The reason for this is that economically developed cities usually have better digital infrastructure and resources, enabling them to promote green development more effectively when implementing digital governance. Economically disadvantaged cities may face technological backwardness and insufficient resources, and thus the impact of digital governance on their green development may not be as significant as in economically developed cities; on the other hand, large cities usually have more resources and technological bases that allow them to implement digital governance and promote green development more effectively. Compared to small- and medium-sized cities, large cities also possess stronger policy implementation and technological innovation capabilities, although they may face more complex environmental and transportation challenges. Heterogeneity analysis can therefore reveal the differentiated effects of digital governance on green development at different levels of economic development and population sizes, leading to more targeted policies.

#### 4.3.1 Heterogeneity analysis of GDP *per capita*

Variations in economic development levels across Chinese cities may impact the efficacy of local government administration, human education levels, and the business environment, thereby influencing the extent to which the program affects green development. Given the widespread recognition of GDP as a measure of economic development, this study categorizes cities into two equal groups based on median per capita GDP (35). These groups are the high per capita GDP group (High GDPPC) and the low per capita GDP group (Low GDPPC). Subsequently, this study investigates the heterogeneity in the impact of the program on green development due to GDP variation. Columns 1 and 2 of Table 5 show the results.

**Table 5:** Heterogeneity analysis results.

	1	2	3	4
	High GDPPC	Low GDPPC	Large cities	Small-medium cities
Variable	GD	GD	GD	GD
DID	−0.043*	−0.002	−0.023**	−0.090
	(0.025)	(0.013)	(0.011)	(0.080)
Controls	YES	YES	YES	YES
Year FE	YES	YES	YES	YES
City FE	YES	YES	YES	YES
Cons	0.0258	0.105***	0.050**	0.168
	(0.040)	(0.019)	(0.023)	(0.101)
*N*	1974	1974	3,117	831
*R* ^2^	0.085	0.323	0.243	0.049

(1) Standard errors in parentheses; (2) *p < 0.1, **p < 0.05, ***p < 0.01.

It can be seen that digital governance significantly boosts local green development in high per capita GDP cities, whereas digital governance hinders local green development in the low per capita GDP cities. A possible reason is that cities with high per capita GDP in China usually possess higher fiscal revenue and technical levels, and can invest more resources in the construction and operation of digital governance infrastructure to better use big data analysis, artificial intelligence, and other technical means to monitor the environment, optimize resource utilization, and effectively promote green development. By contrast, cities with low per capita GDP may struggle with the limited fiscal resources required for the successful implementation of the program. Additionally, residents in these areas may typically receive less education and consequently may not fully appreciate the benefits of a digital environment. This impairs the construction and operation of the digital governance system and hampers its role in fostering green development.

#### 4.3.2 Heterogeneity analysis of city size

Generally, cities of different sizes have significant variations in capital, market, labor, and so on. Heterogeneity may affect the role of digital governance of different cities in promoting green development. Consequently, in terms of the “Notice of the State Council of China on Adjusting the Criteria for the Classification of Urban Scale,” we categorized our sample cities into two groups: large cities with a permanent population of 1 million or more, and small- and medium-sized cities with a permanent population of less than 1 million, and then regressed separately. Columns 3 and 4 of Table 5 display the regression results. The results reveal that the role of digital governance in promoting green development is more significant in large cities than in small-and medium-sized cities. A possible reason is that the digital infrastructure of big cities is relatively perfect, and digital technology can be used more effectively to monitor environmental conditions and resource utilization to better promote green development.

## 5 Further analysis

To delve deeper into the factors that contribute to the finding of the baseline model, we conducted mediating models that elucidate the pathways through which the policy exerts its influence, in accordance with established practices in the existing literature (36, 37). According to the aforementioned research Hypothesis 2, digital governance can promote green development by optimizing the industrial structure. To verify this hypothesis, this study constructed the following mediating effect model:


(4) $$Z_{\mathrm{it}}=\phi_{0}+\phi_{1}DID_{\mathrm{it}}+\mathrm{\phi Control}s_{\mathrm{it}}+\mu_{i}+\nu_{t}+\varepsilon_{\mathrm{it}}$$

where the variable $Z_{\mathrm{it}}$ acts as a mediator for industrial structure optimization, including supererogation and rationalization level of industrial structure. Equation 4 captures the impact of digital governance on the intermediary variable.

The outcomes of the mediating effect test are recorded in Table 6. Column 1 is consistent with the conclusions of the baseline model. In Columns 2 and 3, the regression results for industrial structure supererogation (ISS) and industrial structure rationalization (ISR), correspond to Equation 4.

**Table 6:** Mediating effect test results.

	1	2	3
Variable	GD	ISS	ISR
DID	−0.035**	0.079***	0.196**
	(0.014)	(0.029)	(0.085)
Controls	YES	YES	YES
Year FE	YES	YES	YES
City FE	YES	YES	YES
Cons	0.092***	0.616***	−0.074
	(0.027)	(0.047)	(0.197)
*N*	3,948	3,948	3,948
*R* ^2^	0.102	0.660	0.201

(1) Standard errors in parentheses; (2) *p < 0.1, **p < 0.05, ***p < 0.01.

The results show that digital governance plays a significant role in promoting both supererogation and rationalization of the industrial structure. On the other hand, it has been demonstrated in the literature that the optimization and upgrading of industrial structure can promote green development in cities (38). Thus, digital governance promotes green development by optimizing industrial structure, Hypothesis 2 is verified.

## 6 Discussion

Digital governance has a significant role in promoting urban green development, but the existing literature has not carried out in-depth research in this area. In view of this, based on the data of 282 cities from 2007 to 2020, and using the policy of “pilot cities regarding information for public well-being” implemented in 2014 as a quasi-natural experiment, this paper examines the specific impact of digital governance on green development by constructing double-difference models and mediation effect models. Mechanisms. The results show that digital governance significantly promotes urban green development, which contributes to environmental protection and public health. This is consistent with previous theoretical expectations. Specifically, there is obvious heterogeneity in the impact of digital governance: in economically developed cities, the effect of digital governance is significantly better than that of economically backward cities due to the existence of better digital infrastructure and policy support; in large cities, digital governance promotes green development more significantly due to the scale effect and resource advantage.

These results also indicate that the advanced industrial structure and the rationalization of industrial structure play an important intermediary role in the process of promoting green development by digital governance, which is in line with the theoretical framework about the mechanism of green development by digital governance through the optimization of industrial structure. In conclusion, this study not only verifies the facilitating effect of digital governance on green development, but also reveals its heterogeneous impacts under different city types and economic conditions, as well as the key role of industrial structure in this process.

The limitations of our analysis are as follows. First, it should be noted that the potential bias of this study mainly comes from policy implementation effects vary in different pilot cities. This study has discussed the impact that whether the policies implement or not on urban green development. However, the policy effects in different pilot cities may be inconsistent and even in some cities are relatively delayed. Second, although the sample size is large, it may still be incomplete. For example, this study involves fewer ethnic autonomous areas, and some remote areas are also excluded due to data limitations. Third, since the study is mainly based on a sample of Chinese cities, the applicability of the conclusions to other countries or regions may be limited, especially in places where there are large differences in the level of digital governance, industrial structure, and degree of green development. Therefore, the research findings need to be appropriately adapted and applied.

Based on the limitations of this study, there are following specific areas for future research. First, the time span of the study could be extended to analyze the sustained impact of digital governance on green development over a longer period. By looking at the long-term effects of the programs after implementation, we can explore whether there is a lasting positive effect or whether it diminishes over time. Second, we can compare China’s digital governance with other countries’ practices, especially with the relevant practices in other developing countries, and analyze how the digital governance strategies of different countries affect green development. This can help identify which governance models are more effective and how successful experiences can be scaled up globally. Third, the evolution of digital governance policies at different stages and their impact on green development can be further explored. For example, it could analyze whether new digital governance policies have been introduced since 2014 and their overlapping effect with the program of “pilot cities regarding information for public well-being”.

## 7 Conclusion

Our analysis provides practical implications that can provide valuable guidance for policymakers and urban planners. Cities in China and beyond can consider adopting similar digital governance development initiatives to stimulate green development. It is noteworthy that this impact can be enhanced by optimizing the industrial structure. Digital governance plays a significant role in promoting green transformation of industrial structures. Therefore, the government should establish a digital service platform to provide services such as green industrial policy interpretation, financial support, and approval process optimization to reduce the threshold of entrepreneurship in the green industry. Simultaneously, it is necessary for the government to employ digital technologies such as big data to assess market demand and industrial trends, which can provide market information for firms. In addition, the government should share more data resources with enterprises and research institutions to promote green innovation and cooperation.

In addition, the application of digital governance should be optimized according to the specific conditions of cities at different levels of economic development and city sizes. In economically developed large cities, the resource advantages of digital governance should be fully utilized to promote the application of advanced technologies, such as intelligent traffic management, environmental protection monitoring systems and smart energy management, in order to maximize its contribution to green development. In economically backward or small- and medium-sized cities, digital governance should focus on the construction and optimization of infrastructure, and enhance the efficiency of public services and resource management capabilities through digital platforms, so as to gradually promote the process of green development. At the same time, emphasis should be placed on nurturing digital governance talents and strengthening digital infrastructure construction to narrow the gap with developed regions.

Finally, to further improve relevant policy measures, policymakers should prioritize the promotion of digital green development strategies in economically developed regions, for example, by encouraging enterprises to use big data and Internet of Things technologies to improve resource efficiency and reduce pollution emissions. In small- and medium-sized cities and economically backward regions, the government can enhance the transparency and efficiency of public services through digital platforms and promote the popularization and implementation of green projects. In addition, policies should support the optimization and upgrading of the industrial structure, and promote the transformation of the green economy and the sustainable development of cities by encouraging the research and development of green technologies, supporting the development of green industries, and optimizing the industrial layout using digital governance tools.

## Data Availability

The original contributions presented in the study are included in the article/supplementary material, further inquiries can be directed to the corresponding author.

## Footnotes

^1^This number of cities is relatively complete data available in China at present, and most other existing studies are based on this number.
